# Multi‐omics reveal the gut microbiota‐mediated severe foraging environment adaption of small wild ruminants in the Three‐River‐Source National Park, China

**DOI:** 10.1111/1749-4877.12830

**Published:** 2024-05-02

**Authors:** Hongjin LIU, Xinquan ZHAO, Shixiao XU, Liang ZHAO, Xueping HAN, Xianli XU, Na ZHAO, Linyong HU, Chongliang LUO, Xungang WANG, Qian ZHANG, Tongqing GUO

**Affiliations:** ^1^ Key Laboratory of Adaptation and Evolution of Plateau Biota, Northwest Institute of Plateau Biology Chinese Academy of Sciences Xining Qinghai China; ^2^ Institute of Sanjiangyuan National Park Chinese Academy of Sciences Xining China; ^3^ Sanjiangyuan Grassland Ecosystem National Observation and Research Station Xining China; ^4^ University of Chinese Academy of Sciences Beijing China; ^5^ Technology Extension Service of Animal Husbandry of Qinghai Xining China; ^6^ State Key Laboratory of Plateau Ecology and Agriculture Qinghai University Xining China

**Keywords:** adaptability, gut microbiota, multi‐omics sequencing, small ruminants, Three‐River‐Source National Park

## Abstract

The Tibetan antelope (*Pantholops hodgsonii*), blue sheep (*Pseudois nayaur)*, and Tibetan sheep (*Ovis aries*) are the dominant small ruminants in the Three‐River‐Source National Park (TRSNP). However, knowledge about the association between gut microbiota and host adaptability remains poorly understood. Herein, multi‐omics sequencing approaches were employed to investigate the gut microbiota‐mediated forage adaption in these ruminants. The results revealed that although wild ruminants (WR) of *P*. *hodgsoni* and *P. nayaur* were faced with severe foraging environments with significantly low vegetation coverage and nutrition, the apparent forage digestibility of dry matter, crude protein, and acid detergent fiber was significantly higher than that of *O. aries*. The 16s rRNA sequencing showed that the gut microbiota in WR underwent convergent evolution, and alpha diversity in these two groups was significantly higher than that in *O. aries*. Moreover, indicator species, including Bacteroidetes and Firmicutes, exhibited positive relationships with apparent forage digestibility, and their relative abundances were enriched in the gut of WR. Enterotype analysis further revealed that enterotype 1 belonged to WR, and the abundance of fatty acid synthesis metabolic pathway‐related enzyme genes was significantly higher than enterotype 2, represented by *O. aries*. Besides, the metagenomic analysis identified 14 pathogenic bacterial species, among which 10 potentially pathogenic bacteria were significantly enriched in the gut microbiota of *O. aries*. Furthermore, the cellulolytic strains and genes encoding cellulase and hemicellulase were significantly enriched in WR. In conclusion, our results provide new evidence of gut microbiota to facilitate wildlife adaption in severe foraging environments of the TRSNP, China.

## Graphical abstract

This research reveals the gut microbiota‐mediated severe foraging environment adaptation of wild small ruminants in the Three‐River‐Source National Park. Unlike Tibetan sheep (TS), blue sheep (PN and Tibetan antelope (TA) often grazed low‐quality herbage in a wildness environment, and the higher microbial diversity and resilient network characteristics enabled the hosts to cope with flexible feeding environments. Environmental heterogeneity made the gut microbiota of PN and TA exhibit convergent evolutionary characteristics to adapt to nutritional stress from the habitats. Moreover, a study on microbial enterotypes revealed the two enterotypes, especially E1 exhibited by PN and TA, exhibit higher forage utilization potential and typical microorganisms involved in fatty acid and oxidative phosphorylation pathways play vital roles in energy production and maintaining the hosts’ energy balance in the face of cold, anoxia, and food scarcity situation. Furthermore, the less abundant the microbial pathogens, the more abundant the forage‐degrading microbiota, and the CAZymes genes encoding cellulose and hemicellulase enable wild small ruminants to better adapt to low quantity and poor‐quality forage environments.

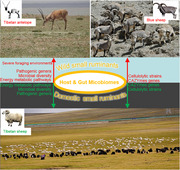

## INTRODUCTION

The Three‐River‐Source National Park (TRSNP) is located on the Qinghai–Tibetan Plateau (QTP), “The roof of the world,” with an area of 19.07 km^2^ and is an ecological unit with unique geological, geographical, and resource features (Liu *et al.* 2022). Owing to the presence of various mountains and water resources, it acts as a “Noah's Ark” for wildlife reproduction and serves as a biological gene bank with significant scientific research value (Zhang *et al.* 2023). The harsh high‐altitude (average 4500 m) conditions with severe cold, hypoxia, and strong ultraviolet light also make TRSNP a natural laboratory for studying the adaption and evolution of animals (Liu *et al.* 2022; Zeng *et al.* 2022). Gut microbiota act as the host's “second genome,” form an inseparable relationship with the host (Zhu *et al.* 2010), and play a crucial role in digestion (Peng *et al.* 2021), immune maintenance (Gensollen *et al.* 2016), and metabolic homeostasis (Buffie & Pamer 2013). As an essential “acquired organ” in animals, the gut microbiota are also an important element for studying the interactions between animals and their environment adaption and evolution and, thus, has attracted increasing attention (Nicholson *et al.* 2012; O'Toole & Jeffery 2015).

Tibetan antelope (TA; *Pantholops hodgsonii*), blue sheep (PN; *Pseudois nayaur*), and Tibetan sheep (TS; *Ovis aries*) are three typical small ruminants that are widely distributed in the TRSNP (Zhang *et al.* 2023). However, low vegetation cover and long periods of grass withering (approximately 8 months per year) are crucial constraints for these herbivores living in such high‐altitude areas (Yifan *et al.* 2008). Small ruminants, especially wild ruminants (WR), require highly efficient energy extraction and absorption mechanisms to balance survival threats in such extreme environments. For example, the ability of TA to combat adverse environmental effects has been thoroughly investigated in terms of physiology, cell biology, and molecular biology (Rong *et al.* 2012; Ge *et al.* 2013). In terms of microbiology, it is well established that herbivores convert the indigestible forage substances cellulose and hemicellulose into short‐chain fatty acids, which can be digested and absorbed by the hosts mainly through interactions with the gastrointestinal microbiota (Flint *et al.* 2008; Zhang *et al.* 2016). However, existing studies on the function of diet‐driven gastrointestinal microbiota of TA (Bai *et al.* 2018; Shi *et al.* 2021; Shang *et al.* 2022) and PN (Chi *et al.* 2019; Sun *et al.* 2019; Zhu *et al.* 2020; Zhao *et al.* 2023) only focused on the basic level of microbial composition and diversity based on 16S rRNA gene sequencing. With the development of microbial sequencing, multi‐omics techniques have become invaluable for unveiling the comprehensive interaction mechanism between host and environment.

Hence, in this study, 16S rRNA and metagenomic sequencing were conducted to investigate the functional capacity of gut microbiota and their co‐evolution with hosts during their adaptation to severe foraging environments among TA, PN, and TS. Unlike TS, which are under the care of herders year‐round, TA and PN have to forage on their own in a complex and ever‐changing natural environment. In addition, they often face survival pressures, such as predation and competition, owing to insufficient food supplies. Therefore, we hypothesized that the gut microbiota of PN and TA have a more efficient microbial‐mediated forage utilization mechanism than that of TS, which enables the hosts to cope with harsh foraging environments.

This study aimed to clarify the co‐evolution of gut microbiota and hosts in the TRSNP. These results may provide new evidence for understanding the roles of gut microbial‐mediated severe foraging environment adaption in small wild ruminants in the Three‐River‐Source National Park, China.

## MATERIALS AND METHODS

### Sampling sites

**Figure 1:**
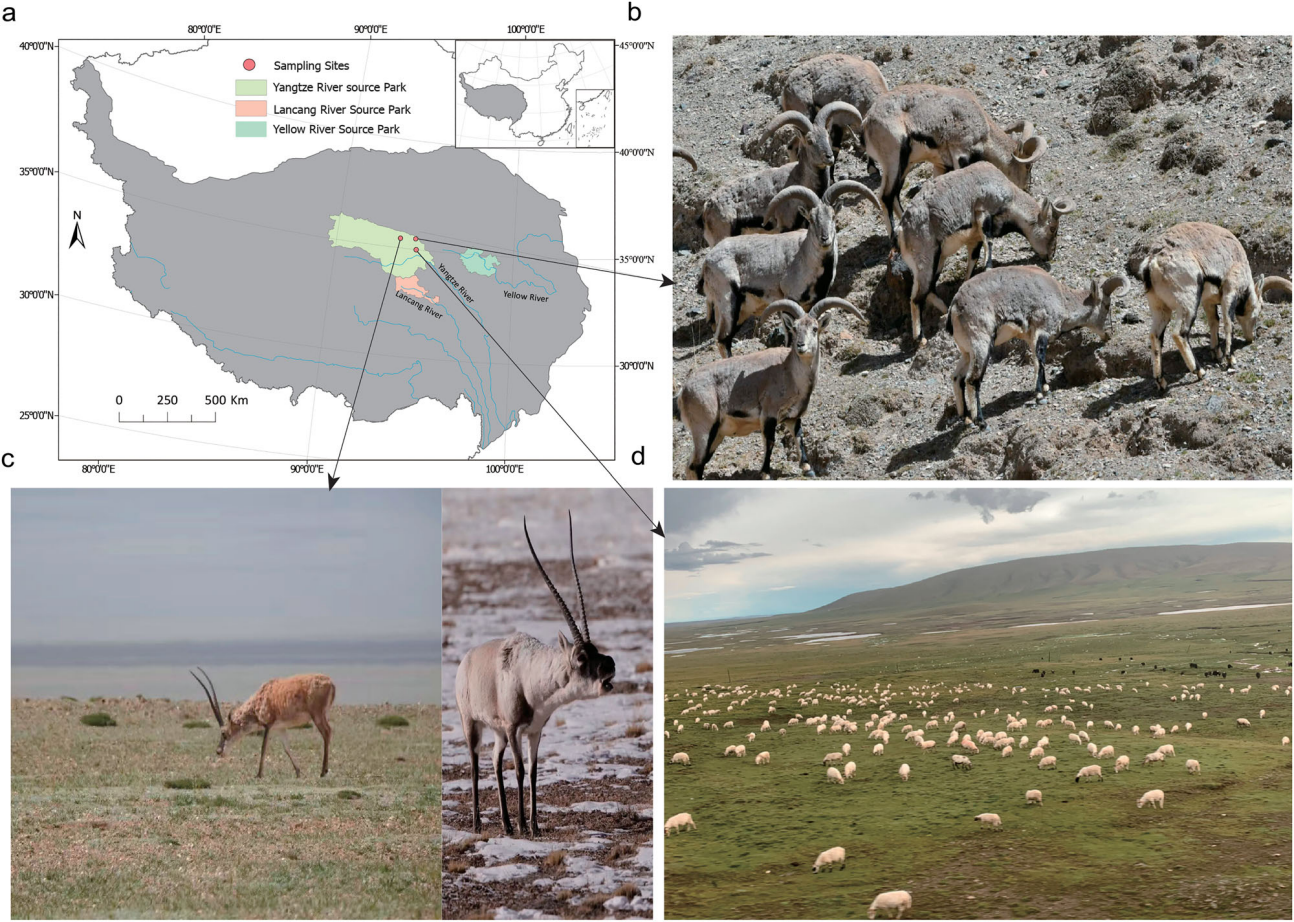
Sampling site and experimental animal habitat condition information. (a) The sampling sites in the Three‐River‐Source National Park. (b) Small herds of blue sheep leisurely feed on the sparse grass on steep hills. (c) The photograph of a Tibetan antelope (TA) munching on grass in the desertified grassland of Hoh Xil in summer (left). A TA looks out onto a snow‐covered meadow (right) on a cold winter day. (d) Tibetan sheep feed on an abundant alpine meadow under the care of herdsmen.

Fresh fecal samples were collected in August 2018 from the TRSNP. TS fecal samples were collected near the Three‐River‐Source Alpine Meadow Integrated System Observation of the Chinese Academy of Sciences in Yangtze River Source Park (Qumarleb County, Yushu Prefecture, Qinghai Province, China; 34°59′36″N, 94°29′20″E; altitude 4350 m). Fecal samples of TA and PN were collected in the Hoh Xil area (35°26′28″N, 93°32′27″E, altitude 4470 m and 35°53′8″N, 94°23′12″E, altitude 4310 m, respectively) (Fig. 1). None of the animals received concentrate supplementation.

### Forage and fecal sample collection

Herbage collection methods used in this study were similar to those reported by Liu *et al.* (2022). Briefly, ten 50 cm × 50 cm quadrat frames were applied to collect the above‐ground biomass in each experimental site. The ocular method was used to estimate herbage coverage. Different types of plants were measured in a quadrat frame, and the average height was taken to represent community height.

Owing to the high alertness of animals during the sampling process, we observed the hosts from a distance with a binocular telescope (Yunnan North Optical & Electron Group Co. Ltd., China). After animals left the defecation area, fresh fecal samples of different animals were collected immediately. To avoid cross‐infection, each sample was collected using autoclaved disposable polyethylene gloves. Approximately 2 g of each sample was collected in cryotubes and labeled. Nineteen fresh fecal samples were collected from 8 TS, 6 TA, and 5 PN. All samples were promptly stored in liquid nitrogen and were later transferred to a −80°C ultra‐low temperature refrigerator until subsequent high‐throughput sequencing.

### Determination of forage nutrition composition and the hosts’ apparent nutrient digestibility

The dry matter (DM, Method No. 2001.12), crude protein (CP, Method No. 984.13), and ether extract (EE, Method NO. 954.02) contents were determined by using AOAC methods (Feldsine *et al.* 2002). Natural detergent fiber (NDF) and acid detergent fiber (ADF) contents were analyzed according to Van Soest *et al.* (1991). Apparent nutrient digestibility was determined using the acid‐insoluble ash method as described by Liu *et al.* (2022).

### DNA extraction, PCR amplification, and 16S rRNA sequencing

The microbial DNA from ∼0.2 g fecal samples was extracted using an E.Z.N.A.® Stool DNA Kit (Omega Bio‐tek, Norcross, GA, USA) according to the manufacturer's protocols. The concentration and quality of the extracted DNA were assessed by OD 260/OD 280 ratios using a NanoDrop ^TM^ 2000 (Thermo Fisher Scientific, Waltham, MA, USA), followed by integrity evaluation using 2% agarose gel electrophoresis. The universal primers 341F (5ʹ‐CCTACGGGNGGCWGCAG‐3ʹ) and 806R (5ʹ‐GGACTACHVGGGTATCTAAT‐3ʹ) were employed to amplify the V3‐V4 hypervariable region of 16S rRNA genes of the purified DNA. Barcodes consisted of six‐base sequences unique to each sample were added. A 20‐μL PCR amplification mixture was prepared for each sample, containing 4 μL of 5 × FastPfu Buffer, 2 μL of 2.5 mM dNTPs, 0.8 μL of each primer (5 μM), 0.4 μL of FastPfu Polymerase, and 10 ng of gut microbial DNA. The following PCR reaction conditions included an initial denaturation at 95°C for 2 min, followed by 25 cycles at 95°C for 30 s, annealing at 55°C for 30 s, and a final extension at 72°C for 5 min. The PCR products were detected on 2% agarose gels, purified using the AxyPrep DNA Gel Extraction Kit (Axygen Biosciences, Union City, CA, USA) according to the manufacturer's protocol, and quantified using QuantiFluor^TM^–ST (Promega, Wisconsin, USA). For library construction, the purified PCR products were quantified using Qubit^®^3.0 (Life Invitrogen, State of California, USA), and the quantity checking was used by Agilent 2100 Bionalyzer system (Agilent Technologies, USA). All amplicons were pooled into equimolar sample concentrations. After the quality appraisal, the amplicon library was sequenced on an Illumina Hiseq2500 platform (Nanjing GenePioneer Co. Ltd), and 250‐bp long paired‐end reads were generated.

Bioinformatic analysis of the library sequence was performed using EasyAmplicon (https://github.com/YongxinLiu/EasyAmplicon). Briefly, the paired‐end sequences were merged, quality filtered, and dereplicated with the running codes –fastq_mergepairs, –fastx_filter, and –derep_fullength, respectively, using the default parameters in VSEARCH v.2.15.2 (https://github.com/torognes/vsearch) (Rognes *et al.* 2016). Thereafter, the non‐redundant sequences were denoised into amplicon sequence variants (ASVs) with the unoise3 algorithm implemented in USEARCH v.10.0.24 (http://www.drive5.com/usearch/; Edgar 2010), and a feature table was generated with the –usearch_global function. As chloroplast DNA sequences can exist in the feature table, EasyAmplicon's built‐in script (otutab_filter_nonBac.R) was used to remove the chloroplast sequences. Finally, the sintax algorithm in USEARCH, based on the Ribosomal Database Project classifier v.16, was employed for the taxonomic classification of ASVs. To standardize the sampling efforts across samples, each sample was rarefied to the same number of reads (36 000) to assess the alpha diversity using the Shannon diversity and Chao1 richness indices.

### Meta‐genomic sequencing, assembly, and annotation

The microPITA (microbiomes Picking Interesting Taxonomic Abundance) (Tickle *et al.* 2013) analysis was used to select typical samples based on the term “most dissimilar,” “most representative,” “maximum diversity,” and “multiple selections” according to 16s rRNA sequencing profiles. Finally, five TS fecal samples, five PN fecal samples, and six TA samples were selected for the follow‐up shortgun metagenomics analysis (Fig. S1, Supporting Information). The genomic DNA was extracted by the CTAB method (Barbier *et al.* 2019). The genomic DNA quality control, PCR amplification, and library preparation were carried out based on the method by Liu *et al.* (2022). Metagenomic sequencing was performed on an Illumina HiSeq 2500 platform (Novogene Biological Information Technology Co., Beijing, China), and 150‐bp paired‐end reads were generated. The detailed bioinformatics analysis methods have been described previously by Liu *et al.* (2022). Briefly, bowtie2 (https://www.encodeproject.org/software/bowtie2/) and SOAPdenovo (https://github.com/aquaskyline/SOAPdenovo2) were used to obtain high‐quality clean reads and scaffolds (Karlsson *et al.* 2012; Luo *et al.* 2012). MetaGeneMark v.2.1.0 (https://github.com/gatechgenemark/ MetaGeneMark‐2) was used to predict the contigs (≥500 bp) in each sample (Nielsen *et al.* 2014). The open reading frames were clustered into a non‐redundant dataset using CH‐HIT (Li & Godzik 2006). DIAMOND (https://github.com/bbuchfink/diamond) was used to align the unigenes with the bacteria and archaea extracted from the NR database v.2018.01 for taxonomic profile annotation. For gene function annotation, the unigenes were blasted using CAZy, the carbohydrate‐active enzymes (CAZymes) database (Drula *et al.* 2022) and the Kyoto Encyclopedia of Genes and Genomes (KEGG) (Kanehisa *et al.* 2017).

### Enterotype clustering

For distinguishing the distinct mammalian gut microbial community composition types, termed enterotypes, we employed the enterotype clustering method (Arumugam *et al.* 2011; Wu *et al.* 2011; Costea *et al.* 2018) to evaluate whether the gut microbiota divided into clusters with functional properties that varied among hosts. For all datasets, root Jensen–Shannon divergence and Bray–Curtis dissimilarity values were calculated from genus‐level relative abundance profiles in R v.4.2.1, as described by Guo *et al.* (2021). The optimal number of clusters (*k*) for each dataset was determined using the Calinski–Harabasz index, and the average silhouette score was calculated using *clustersim* v.2.1.4 and *cluster* v.0.50.1 packages, for Bray–Curtis and root Jensen–Shannon divergence metrics, respectively, in R. Enterotype clustering of the gut microbiota was assigned to *k* clusters for each dataset using the partitioning around medoids algorithm implemented in the *cluster* package. In terms of the Calinski–Harabasz index, the distribution map of the *k* value obtained based on the root Jensen–Shannon divergence algorithm was more consistent with the estimated calculation. Hence, we adopted the Jensen–Shannon divergence algorithm for the subsequent enterotype analysis. Simper analysis was conducted using the *simper* function in the *vegan* v.2.6.4 package to cumulate the contribution of each genus to the intestinal types.

### Molecular ecological network construction

Co‐occurrence networks were constructed based on the random matrix theory approach (Yuan *et al.* 2021) using the online Molecular Ecology Networks Analysis pipeline (http://ieg2.ou.edu/MENA), and the detailed operational procedures have been described elsewhere (Liu *et al.* 2022). This pipeline has a strict requirement for the number of samples in each group, and only groups with eight or more samples can be used for network construction. To enhance the precision of molecular network construction, we merged the PN and TA groups to form the wild rumimant groups (WR) based on their similar microbial community structure and shorter phylogenetic distance and compared them with the TS group to analyze the similarities and dissimilarities of the gut microbiota molecular network between wild and domesticated ruminants. To guarantee the reliability of the Pearson correlation calculation, only the ASVs present in more than half of the samples were selected for network construction. Network topological properties including average connectivity (avgK), average clustering coefficient (avgCC), and average path distance (GD) were calculated. Networks were visualized using the Cytoscape software (v.3.3.0).

### Statistical analysis

In this study, TimeTree online tool (http://www.timetree.org/) was employed to evaluate the species evolution time of PN, TA, and TS. R v.4.2.1 was used for the significance analysis, and Sankey plots or boxplots were generated to visualize the relative abundances of microbial taxa among hosts. The Shapiro–Wilk normality test was implemented using the R function *shapiro.test* to test the normality of the data. The *aov* function in the *multicomp* package v.1.4.20 was used to conduct a one‐way analysis of variance (one‐way ANOVA) on the forage nutrition composition, apparent nutrient digestibility, and alpha diversity of gut microbiota in different hosts, and the *tukeyHSD* function was used for multiple comparisons. Data that did not conform to a normal distribution were analyzed using the Kruskal–Wallis (multiple groups) and Dunnett's *post hoc* (two‐group comparisons) tests. In addition, Mann–Whitney U tests were used to evaluate changes in gut microbiota taxonomic abundances associated with various enterotypes. To assess whether the beta diversity of gut microbiota communities differs among different ruminants more intuitively, the unweighted pair group method with arithmetic mean (UPGMA) was implemented using the *upgma* function in the *phangorn* package to generate two different types of phylogenetic trees (cladogram and phylogram, respectively). The homogeneity of dispersions for the diversity metric was then tested to identify differences in variance among treatments. Significant differences were determined using 999 permutations of the *betadisper* function in the *vegan* package v.2.6.4 of R. To identify the microbial taxa that best represented variation across different hosts, indicator analysis was conducted using the *indicspecies* package v.1.7.12 in R. A Mantel test with 999 permutations was used to calculate the correlations between forage nutrient digestibility and the indicator species by using the LinkET package of R (https://github.com/Hy4m/linkET). The r. g. function was used to determine correlations between the binary vectors (Liu *et al.* 2022). Metastats analysis from mothur software v.1.48.0 was used to detect the different carbohydrate‐metabolizing genes among TA, PN, and TS.

## RESULTS

### Assessment of the forage community characteristics and nutrition composition

As shown in Table 1, the main plant‐based diet for TS was alpine meadows, covered with *Kobresia pygmaea*, *K. humilis*, and *K. capillifolia*. TA includes desertified grasslands covered with *Stipa purpurea*, *Poa annua*, and *Carex moorcroftii*. PN mainly feeds on small shrubs such as *Potentilla fruticosa*, moss, and *Rhododendron* scrub. The herbage average height among different vegetation types were small shrubs > desertified grasslands > alpine meadows (*P* < 0.05). The herbage vegetation coverage, DM contents, CP, and EE in the PN and TA groups were significantly lower than TS (*P* < 0.05). NDF and ADF contents in PN and TA were significantly higher than TS (*P* < 0.05).

**Table 1:** The vegetation community characteristics and nutrition composition in different habitats

	Groups			
Items	TS	PN	TA	*P*‐values
Characteristics of herbage community				
Vegetation type	Alpine meadows	Small shrubs	Desertified grasslands	
Dominant species of herbages	*Kobresia pygmaea*, *Kobresia humilis*, *Kobresia capillifolia*	*Potentilla fruticosa*, Moss, *Stipa capillata*	*Stipa purpurea*, *Poa annua*, *Carex moorcroftii*	
Community height (cm)	5.13 ± 0.26^b^	9.45 ± 0.20^a^	7.26 ± 0.15^a^	<0.05
Vegetation coverage (%)	76.58 ± 1.66^a^	45.29 ± 2.17^b^	41.05 ± 1.15^b^	<0.05
Dry matter contents (g m^−2^)	175.26 ± 6.37^a^	75.66 ± 1.44^a^	78.36 ± 1.05^b^	<0.05
Nutrition composition (%)^†^				
DM	90.52 ± 0.41^b^	95.18 ± 0.53^a^	95.62 ± 0.74^a^	<0.05
CP	12.73 ± 0.70^a^	7.72 ± 0.61^b^	8.19 ± 0.36^b^	<0.05
EE	2.02 ± 0.06^a^	1.84 ± 0.05^b^	1.71 ± 0.03^b^	<0.05
NDF	25.82 ± 0.61^b^	33.41 ± 0.22^a^	32.15 ± 0.68^a^	<0.05
ADF	49.52 ± 1.76^b^	59.66 ± 1.07^a^	62.35 ± 1.49^a^	<0.05

^†^ The nutrition composition is based on dry matter basis. Values with different small letter superscripts in the same row mean significant difference. DM, dry matter; CP, crude protein; EE, ether extract; NDF, neutral detergent fiber; ADF, acid detergent fiber; TS, Tibetan sheep; PN, blue sheep; TA, Tibetan antelope.

### Similarity and dissimilarity of the gut microbiota among TA, PN, and TS

**Figure 2:**
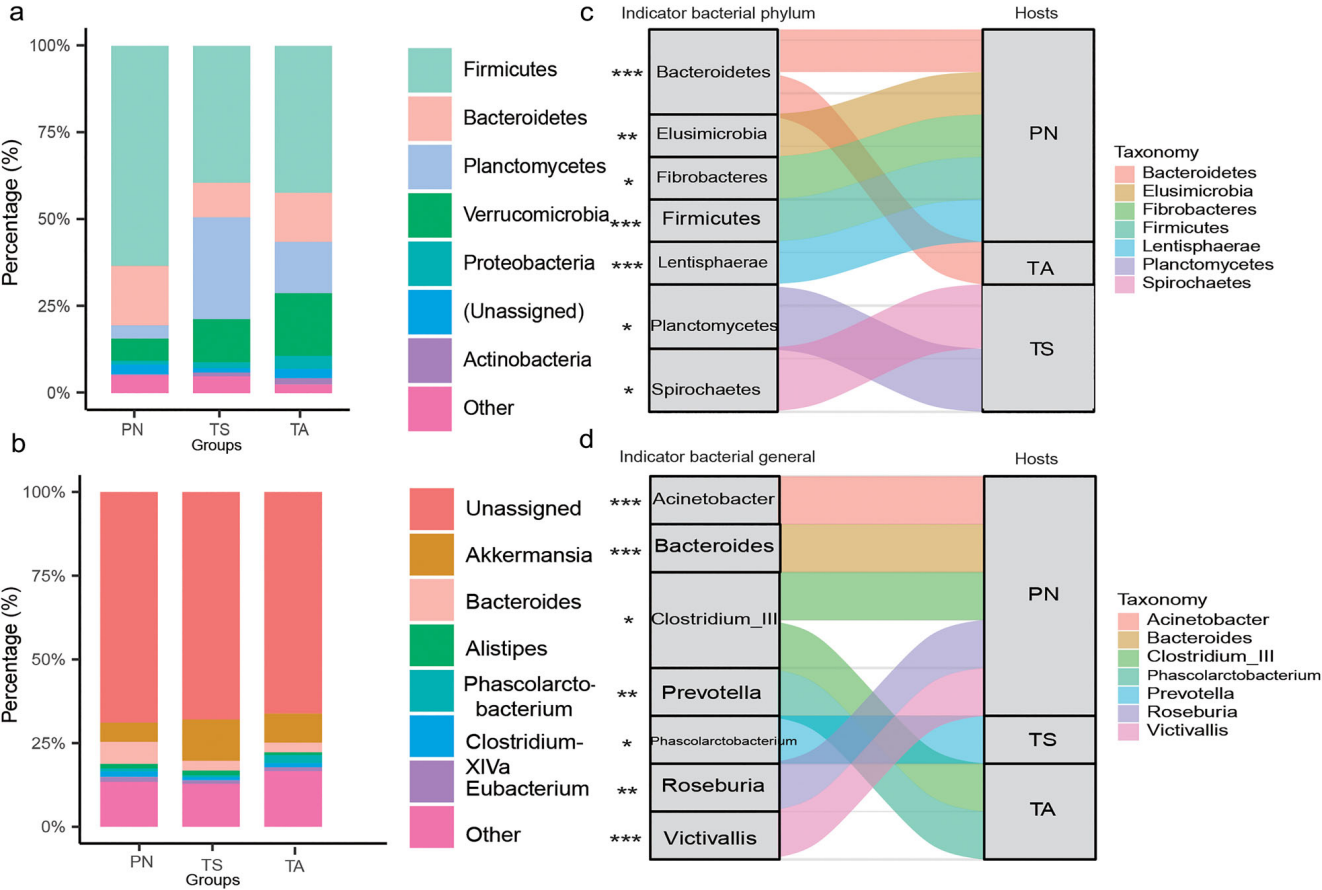
The relative abundances of gut microbiota and indicator taxonomy species among different host groups. The relative abundance of the six most abundant phyla (a) and genera (b) are depicted and colored on stack graphs. Indicator taxonomic analysis of the phyla (c) and genera (d) related to each host are traced by Sankey plots. Lines with different colors and widths represent associations between indicator taxa and hosts. The wider the line, the larger the indicator value. Higher indicator values signify stronger associations between host species and microbial taxa. Indicator values are shown in Fig. S2, Supporting Information. Statistical *P*‐values mean the significant association between taxa and hosts. ^*^ *P* < 0.05, ^**^ *P* < 0.01, ^***^ *P* < 0.001.

16s rRNA sequencing yielded 1 910 442 high‐quality reads after the removal of singleton and chloroplast sequences. A total of 10 527 ASVs were identified (Table S1, Supporting Information), of which 47.84% (of the total abundance, on average) were associated with Firmicutes, 16.84% with Planctomycetes, 13.79% with Verrucomicrobia, and 11.98% with Bacteroidetes, followed by Proteobacteria (2.31%), Actinobacteria (1.09%), and other rare phyla (relative abundance <1%) (Fig. 2a; Table S2, Supporting Information). Similarities in the gut microbiota were also present at the genus level in both TS and wild hosts (PN and TA), with *Akkermansia* (8.23% of the total average), *Bacteroides* (4.09%), and *Phascolarctobacterium* (1.35%) being the most abundant, accounting for 18.41% of the total annotated genera (Fig. 2b and Table S3, Supporting Information).

Indicator taxonomic analyses at the phylum (Fig. 2c) and genus (Fig. 2d) levels revealed differences between the different host groups. In the TA group, indicator species at the phylum and genus levels were Bacteroidetes, *Clostridium_III*, and *Prevotella*. In the TS group, indicator species included Planctomycetes, Spirochaetes, and *Phascolarctobacterium*. In the PN group, the indicators were Bacteroidetes, Elusimicrobia, Fibrobacteres, Firmicutes, Lentisphaerae, *Acinetobacter*, *Bacteroides*, *Clostridium_III*, *Roseburia, and Victivallis*. Notably, the indicator species (phyla: Firmicutes and Bacteroidetes; genera: *Bacteroides* and *Victivallis*), which had higher indicator values, were more abundant in the PN group than in the TA and TS groups (Fig. S2, Supporting Information).

**Figure 3:**
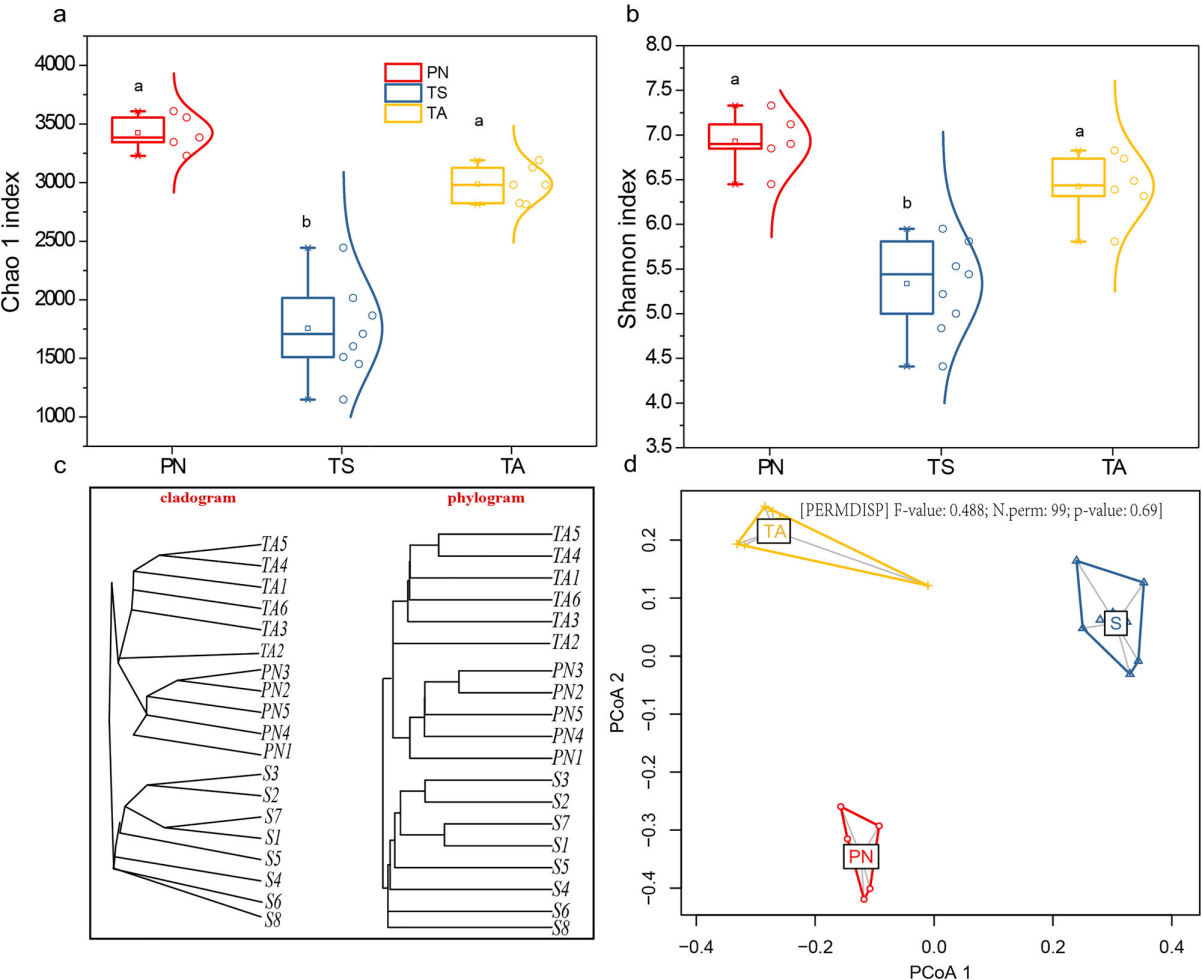
Diversity metrics of gut microbiota among blue sheep (PN), Tibetan sheep (TS), and Tibetan antelope (TA). Chao1 richness index (a) and Shannon diversity index (b) are represented by boxplots. Unweighted pair group method with arithmetic mean (UPGMA) cluster analysis (c) is shown as the cladogram (left) and phylogram (right), respectively. The homogeneity of group dispersion (d) was also tested using permutational analysis of dispersion (PERMDISP). The boxes represent the median (center line) and interquartile ranges; boxes near the median represent the mean. Different letters denote statistically significant differences at *P* < 0.05.

The dissimilarity in the gut microbiota of PN, TA, and TS was also manifested in alpha and beta diversity (Fig. 3). Both the Chao1 richness indices (Fig. 3a) and Shannon diversity (Fig. 3b) in PN and TA were significantly higher than that in TS (*P* < 0.05). Clustering analysis based on Bray–Curtis dissimilarity matrix (Fig. 3c) revealed the abundance‐based gut microbiota in TA and PN had a closer distance distribution than that in TS, indicating that the microbiota community structures of TA and PN were more similar. The significant differences in microbial community structure were also confirmed through the homogeneity of dispersions test (PERMDISP, *F* = 0.488, *P* = 0.69) as shown in Fig. 3d.

### Apparent nutrient digestibility of forage and their correlations with gut microbiota communities

As shown in Table 2, the apparent nutrient digestibility of DM, CP, and ADF in PN and TA was significantly higher than that of TS (*P* < 0.05). No significant differences were detected for NDF digestibility (*P* = 0.32).

**Table 2:** The apparent nutrient digestibility across different hosts

	Groups			
Items	TS	PN	TA	*P‐*values
Apparent nutrient digestibility				
DM	71.47 ± 1.57^b^	80.48 ± 1.99^a^	79.14 ± 2.16^a^	<0.05
CP	69.61 ± 1.09^b^	82.16 ± 1.19^a^	81.36 ± 7.44^a^	<0.05
NDF	59.20 ± 1.29	68.74 ± 1.62	64.14 ± 4.41	0.32
ADF	51.01 ± 0.86^b^	62.88 ± 1.73^a^	62.04 ± 2.16^a^	<0.05

In the same row, values with different letters mean significant difference (*P* < 0.05), while with the same letter or no superscripts mean no significant difference (*P* > 0.05). DM, dry matter; CP, crude protein; NDF, neutral detergent fiber; ADF, acid detergent fiber; TA, Tibetan antelope; TS, Tibetan sheep; PN, blue sheep.

**Figure 4:**
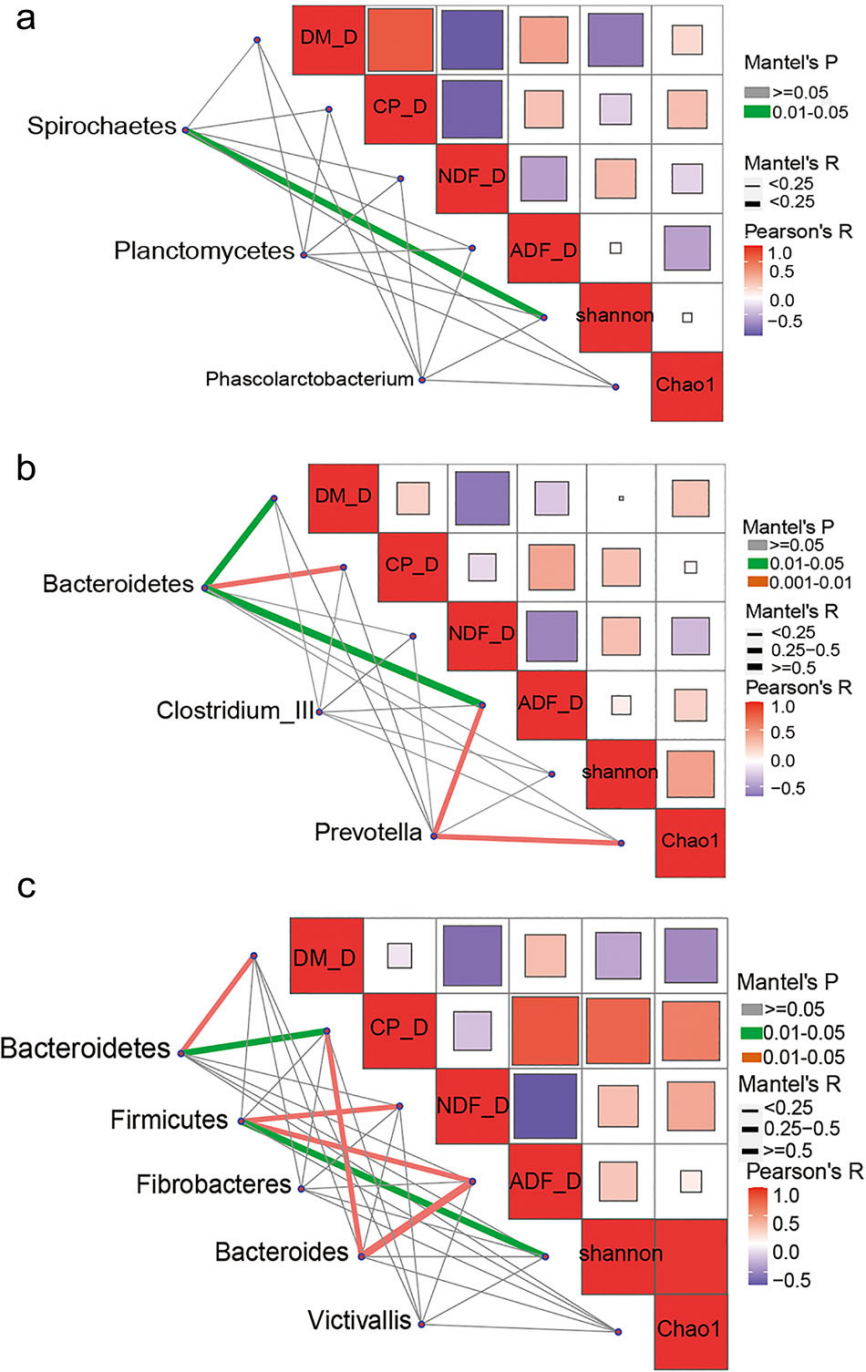
The correlation analysis among forage apparent digestibility, indicator species, and gut microbiota diversity. Panels (a)–(c) are the correlation analysis in Tibetan sheep (TS), Tibetan antelope (TA), and blue sheep (PN) groups, respectively. The size of the squares indicates the value of the pairwise correlation coefficients, and the larger the box, the stronger the correlation. DM_D, dry matter digestibility; CP_D, crude protein digestibility; NDF_D, neutral detergent fiber digestibility; ADF_D, acid detergent fiber digestibility.

To further explore the driving factors that affect the ability of the apparent forage nutrient digestibility, we performed a correlation analysis using the indicator species as the driving factors and the apparent forage digestibility and bacterial alpha diversity as the environmental factors (Fig. 4). In the TS group (Fig. 4a), the Spirochaetes had positive effects on the Shannon indices. In the TA group (Fig. 4b), the relative abundance of the Bacteroidetes had positive effects on the DM, CP, and ADF digestibility. *Prevotella* had positive effects on the forage nutrition digestive ability of the ADF and Chao1 indices; in the PN group (Fig. 4c), both the Bacteroidetes and *Bacteroides* had positive effects on CP digestibility; DM digestibility was affected by the Bacteroidetes as well. The Firmicutes had positive effects on the digestibility of the NDF, ADF, and Shannon indices.

### Gut microbiota enterotype identification and functions of the represented genus

**Figure 5:**
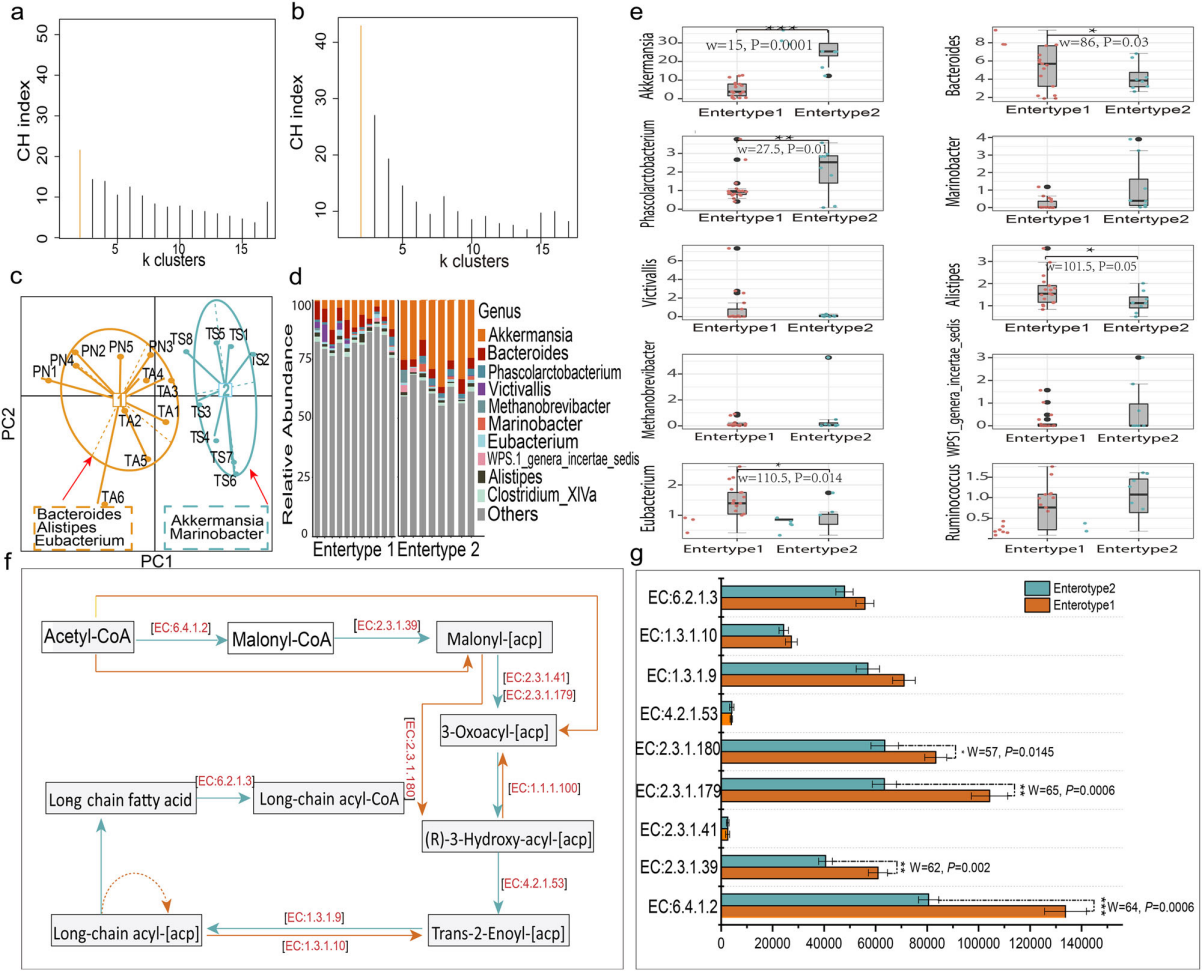
Enterotype distribution and metabolic pathway reconstruction in hosts, associated with the representative genera. Enterotype clusters were identified based on Bray–Curtis dissimilarity (a) and Jensen–Shannon distance (b). Principal coordinate analysis (c) revealed the different enterotype clusters based on Jensen–Shannon distance, according to the relative abundance of gut microbiota at the genus level. (d) The relative abundance of the top 10 genera based on their average contributions to the overall Bray–Curtis dissimilarity. (e) The significance analysis of the top 10 represented genera. (f) The reconstruction of fatty acid synthesis metabolic pathways associated with *Bacterides*, *Alstipes*, *Eubacterium Akkermansia*, and *Marinobacter*. (g) Enzyme abundance significant analysis between E1 and E2. All enzymes and EC numbers were obtained from the Kyoto Encyclopedia of Genes and Genomes (KEGG) database on April 26, 2023. Boxplot center values represent the median, and whiskers represent 0.75 times the interquartile range. All data were evaluated using the Mann–Whitney U test. ^*^ *P* < 0.05, ^**^ *P* < 0.01, ^***^ *P* < 0.001, no ^*^ means no significant difference.

As shown in Fig. 5, the highest CH index values based on partitioning around medoids (Fig. 5a) and root Jensen–Shannon divergence (Fig. 5b) indicated that the gut enterotypes could be classified into two clusters. Principal component analysis further confirmed the optimal number, and the representative hosts and microbial communities of the two enterotypes are shown in Fig. 5c. The gut microbiota present in PN and TA belonged to the enterotype 1 cluster, whereas the gut microbiota of TS belonged to the enterotype 2 cluster. These two clusters were distinguished based on relative differences in their representative bacterial genera. *Bacteroides*, *Alstipes*, and *Eubacterium* were present in enterotype 1, whereas *Akkermansia* and *Marinobacter* were present in enterotype 2 (Fig. 5c). The top 10 relative abundances of representative bacteria (Fig. 5d), *Bacteroides* (W = 86, *P* = 0.03), *Alstipes* (W = 101.5, *P* = 0.01), and *Eubacterium* (W = 110.5, *P* = 0.014), were significantly enriched in enterotype 1. The relative abundances of *Akkermansia* (W = 15, *P* = 0.0001) and *Marinobacter* (W = 27.5, *P* = 0.01) were significantly higher in enterotype 2 (Fig. 5e).

The different distributions of microbial genera allowed us to further evaluate the functional profiles of each enterotype. In this study, we focused on the enzymes involved in fatty acid and energy metabolism under severely cold and sparse vegetation conditions. To explore this, we queried the functional relevance of *Bacteroides*, *Alstipes*, *Eunacterium, Akkermansia*, and *Marinobacter* using the KEGG database. These genera showed convergent enrichment of enzymes involved in fatty acid (FA) biosynthesis (map:00061) pathways (Fig. 5f). Notably, nine enzymes involved in the FA biosynthesis were detected, and their abundance was significantly higher in E1 than in E2 (Fig. 5g). Although no significant differences were observed in the enzymes involved in oxidative phosphorylation (map: map00190), their abundance tended to increase in E1 (Fig. S3, Supporting Information).

### Network analysis of gut microbiota communities

**Figure 6:**
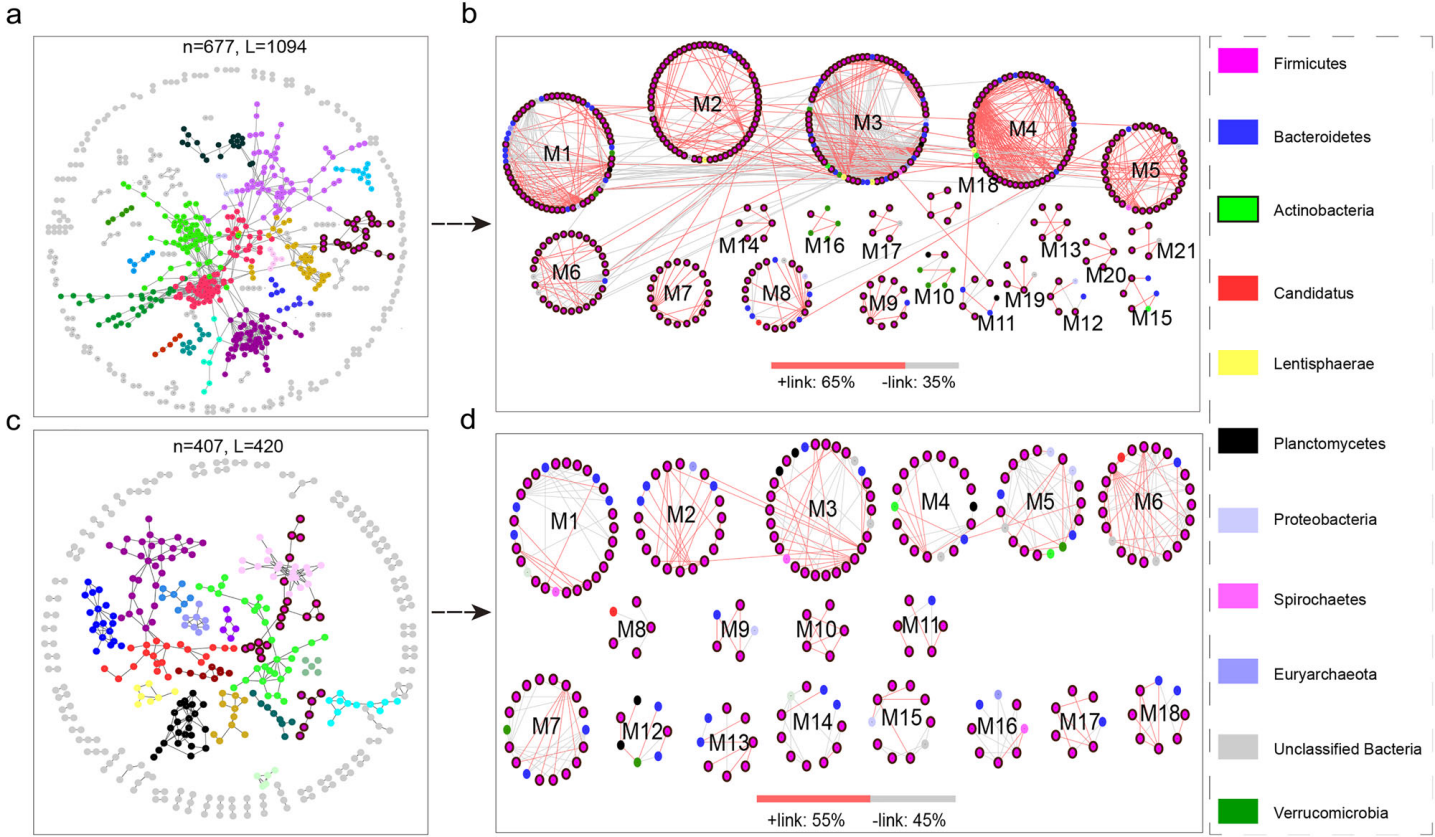
Topological characteristics of the microbial network. Visualization of constructed networks between Tibetan sheep (TS) (a) and wild ruminants (WR) (c). Large modules with ≥5 nodes are shown in different colors, and small modules are shown in gray. Modules preserved in the WR (b) and TS (d) groups. Large modules with ≥5 nodes are presented in a circular layout in the two networks. The colors of nodes in (b) and (d) indicate different taxa and their legends (phylum level) are shown on the right. Red links reveal positive interactions between nodes while gray links indicate negative correlations between nodes. Bars underneath each network indicate the proportions of positive (+) and negative (−) links.

To understand the microbial community interactions of different hosts, a bacterial network was constructed (Fig. 6). The microbial MENs under WR and TS exhibited successional trajectories that differed from the overall distribution (Fig. 6a,c), among which the WR group had more complex and resilient microbial network characteristics, evidenced by higher avgK (3.232) and connectance (0.325) values (Table S4, Supporting Information). Additionally, the WR groups were predominated by higher positive correlation links than TS, implying that mutualism was the prevalent interaction among the gut microbiota of these wild ruminants (Fig. 6b,d). Interestingly, the networks in WR exhibited big‐world characteristics with long geodesics (average shortest path between two nodes) of 6.53 (Table S4, Supporting Information), which reduced the effects of environmental interference throughout the entire network, rendering the entire system more stable and plastic.

### Metagenomic sequencing of the profiles of the potential pathogenic

**Figure 7:**
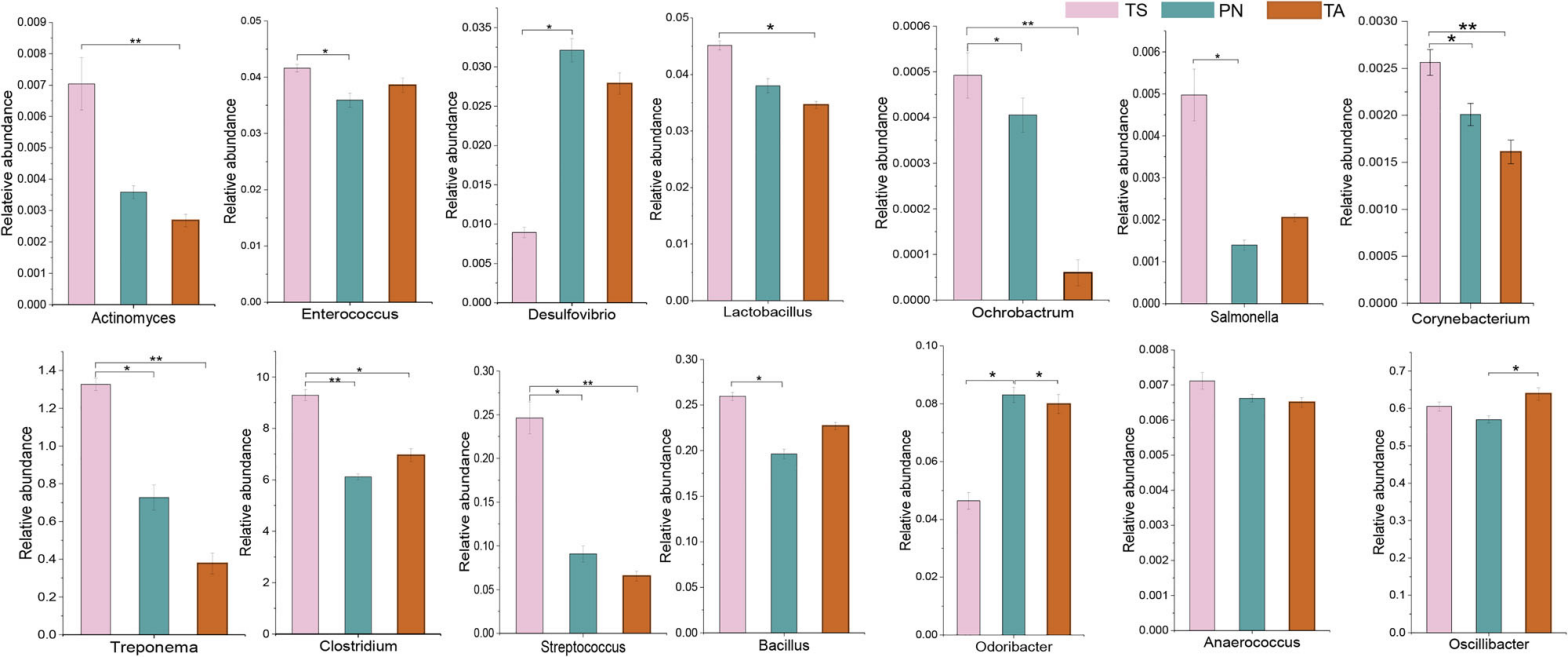
The relative abundance of pathogenic opportunistic bacteria genera among the three small ruminants. The box represents the median (center line) and interquartile ranges, boxes near the median represent the means, and lines near the right of the boxes show the normal distribution of the data. ^*^ *P* < 0.05, ^**^ *P* < 0.01, ^***^ *P* < 0.001. no ^*^ means no significant difference.

We identified 14 pathogenic opportunistic bacterial genera, namely, *Actinomyces*, *Enterococcus*, *Desulfovibrio*, *Lactobacillus*, *Ochrobactrum*, *Salmonella*, *Corynebacterium*, *Treponema*, *Clostridium*, *Streptococcus*, *Bacillus*, *Odoribacter*, *Anaerococcus*, and *Oscillibacter* (Fig. 7). In addition to *Desulfovibrio*, *Odoribacter*, *Anaerococcus*, and *Oscillibacter*, 10 potentially pathogenic bacteria were significantly enriched in the gut microbiota of TS groups (*P* < 0.05).

### Functional microbial species related to cellulose degradation and methane production

**Figure 8:**
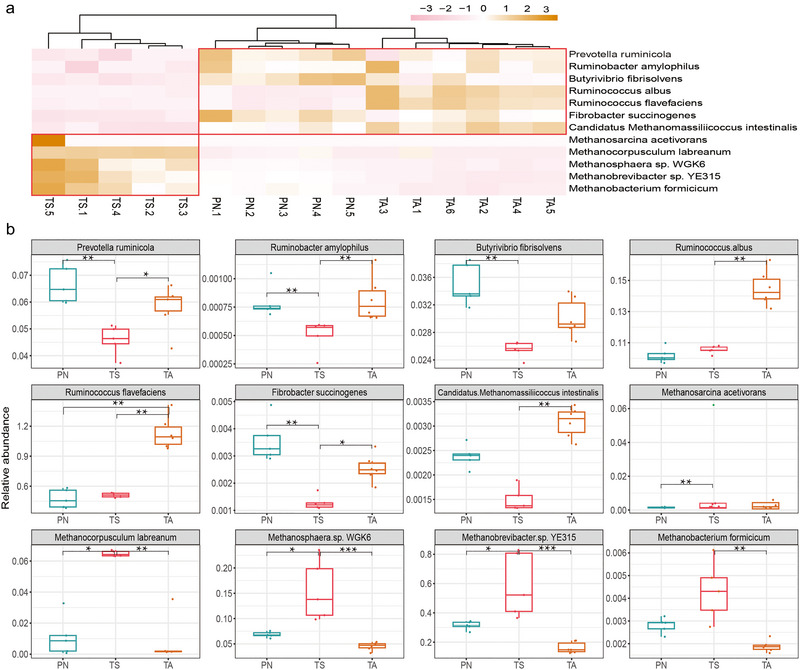
The characteristics of cellulolytic and methanogenic bacteria among the three small ruminants. (a) Heatmap showing the overall distribution of these functional bacteria. (b) The relative abundance of functional bacteria among the blue sheep (PN), Tibetan sheep (TS), and Tibetan antelope (TA) groups. Red boxes in the heatmap show the tendencies for enrichment. All boxplot distributions were obtained from the Kruskal–Wallis (multiple groups) and Dunnett's *post hoc* (two group comparisons) tests with FDR‐corrected *P*‐values, box plot center values that indicate the median, and whiskers represent 0.75 times the interquartile range. ^***^ *P* < 0.001, ^**^ *P* < 0.01, ^*^ *P* <0.05, no ^*^ means no significant difference.

To explore potential cellulose‐degrading and methane‐producing microbial species, 12 main cellulolytic, proteolytic, and methanogenic bacteria were selected according to metagenomic sequencing. The heatmap of the relative abundances of the functional candidate species is shown in Fig. 8a. These candidate species were clustered into two main clusters, among which cellulolytic strains like *Prevotella ruminicola*, *Ruminobacter amylophilus*, *Ruminococcus flavefaciens*, and *Fibrobacter succinogenes* were significantly enriched in the gut of TA and PN, while methanogens such as *Methanocorpusculum labreanum*, *Methanosphaera* sp. WGK6, and *Methanobrevibacter* sp. YE315 were significantly enriched in the TS group (Fig. 8b) (*P <* 0.05).

### Carbohydrate‐metabolizing gene profiles and functions of carbohydrate‐active enzymes

**Figure 9:**
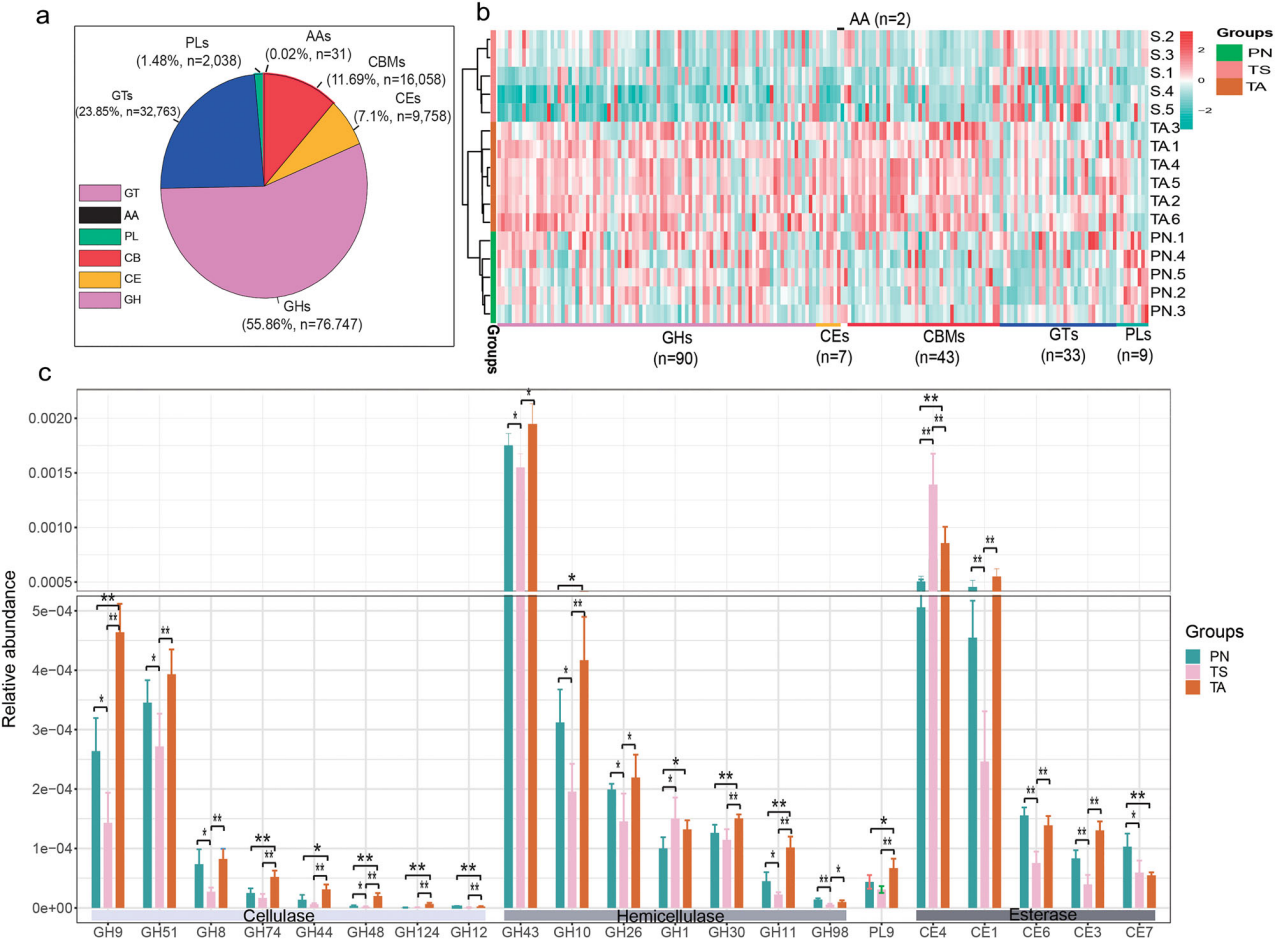
The characteristics of CAZyme genes among different small ruminants. (a) The overall distribution of unigenes. (b) Heatmap visualization of the differential genes after metastats analysis. (c) The significance analysis of unigenes that encode cellulase, hemicellulase, and esterase. The functional classification of unigenes was based on the CAZymes database and the related studies from Gharechahi *et al.* (2021), Peng *et al.* (2021), and Flint *et al.* (2012). ^***^ *P* < 0.001, ^**^ *P* < 0.01, ^*^ *P* <0.05, no ^*^ means no significant difference.

As shown in Fig. 9a, glucoside hydrolases (GHs) were the most abundant enzymes accounting for approximately 55.86% of the enzyme library, followed by glycosyl transferases (GTs, 23.85%), carbohydrate‐binding modules (CBMs, 11.69%), carbohydrate esterases (CEs, 7.1%), polysaccharide lyases (PLs, 1.48%), and auxiliary activities (AAs, 0.02%). Metastats analysis showed 184 differential unigenes, among which GHs were the most abundant (*n* = 90). Most of the unigenes had an obvious enrichment feature that was enriched in the PN and TA groups (Fig. 9b). To further understand the functions of these unigenes, gene function classification was performed according to the CAZy database. Notably, genes encoding cellulases, hemicellulases, and esterases (Fig. 8c) had a significantly higher relative abundance in TA and PN than in TS (*P* < 0.05).

## DISCUSSION

Animals living at high altitudes in the TRSNP are subjected to hypoxia, low temperatures, lower vegetation coverage, and long withering grass periods, which limit the availability of food for wildlife in the area (Lian *et al.* 2007; Zhao *et al.* 2023). The gut microbiota play an important role in converting nutrients into available substances. The abundance of cellulase activity, cellulolytic bacteria, and carbohydrate metabolism genes in the gut of Tibetan wild asses determined their adaptability to the high altitudes of the QTP (Liu *et al.* 2022; Gao *et al.* 2023). Studies on gazelles have found that gut microbiota can improve environmental adaptability by upregulating the expression of metabolic and cellular pathways (Qin *et al.* 2020). TA, PN, and TS are representative wild and domesticated small ruminants in the TRSNP, and research on their gut microbiota can aid in the understanding of the forage nutrition stress adaptation of herbivores.

### Major factors that determine gut microbiota community diversity

Host species, phylogeny, geographical distance, and diet are the main factors that shape the gut microbiota (Sullam *et al.* 2012; Yun *et al.* 2014). In this study, PN and TA, two different species, whose phylogenic split occurred 13 million years ago, exhibited no significant differences in their gut microbial diversity (Fig. S3, Supporting Information). Moreover, the closer abundance‐based gut microbiota distribution distance from phylogenetic analysis revealed the gut microbiota in PN and TA underwent convergent evolution, further indicating that host species and phylogeny may not be influential factors in microbial diversity, which is in accordance with the results of the gut research conducted on wild *Drosophila* (Martinson *et al.* 2017) and Colobine monkeys (Hale *et al.* 2018). Here, the high microbial diversity in PN and TA may be attributed to diet variation. TS are epidemic domestic livestock in the TRSNP, receiving the care of herders, and their feeding environment consists of high‐quality pastures. In contrast, owing to human interference, predators, and low plant coverage, PN and TA have to forage on diverse foods, including lower‐quality indigenous plants on a large spatial scale (Yifan *et al.* 2008). Therefore, we believe that diet variation in the living environment leads to variations in foraging ecology, thereby affecting the structure of the gut microbiota.

### High microbial diversity and complex molecular interactions enable the gut microbiota of wild ruminants to be more resilient to harsh living environments

A high diversity of gut microbes can promote the stability of the gut flora ecosystem and increase the rate of dietary fermentation in the host (Tap *et al.* 2015; Li *et al.* 2019), while indirectly reflecting the poor nutritional status and habit conditions of the host (Liu *et al.* 2019; Yan *et al.* 2022). In our study, the high microbial diversity in PN and TA indicated the gut microbiota in these small wild ruminants were more complex and plentiful. In addition, during the fecal sample collection from the PN population, it was observed that to avoid attacks from natural enemies such as *Panthera uncia*, PN prefer to inhabit areas close to bare rocks and cliffs (Islam *et al.* 2023), consuming plant leaves with a higher cellulose content to sustain survival (Table 1). Meanwhile, due to the perennial drought in Hoh Xil, TA is also facing risks of insufficient nutrition intake. The higher diversity could improve gut microbiota plasticity to cope with the flexible feeding environment.

To further explore the complex interactions occurring in the gut microbiota, such as mutualism, competition, and commensalism (Faust & Raes 2012), we constructed molecular ecology networks (Fig. 6) to investigate the genus interactions according to their mutual exclusion (negative) and co‐occurrence patterns (positive). A positive interaction may result from mutualism or commensalism, while a negative relationship is most likely due to competition, amensalism, predation, and so on (Faust *et al.* 2012). Notably, in our constructed networks, the small wild ruminants were predominated by higher positive correlation links, which revealed that a plant‐based diet in the gut of wild animals might have high efficiency conducive to fermentation through more corporation relationships than that of TS (Li *et al.* 2017; Liu *et al.* 2020b). In addition, we found that in the gut microbiota of the small wild ruminants, the network topological properties of total links, average connectivity, and number of modules were relatively higher than that of TA, indicating that small wild ruminants may harbor a more complex network, particularly a more stable metabolic network for microbial communities against the severe feeding environment.

### Indicator species in small wild ruminants enhance the utilization potential of roughage

We systematically compared the composition and differences of the gut microbiota among the three small ruminants. Similar to the gut microbiota of herbivores such as yaks and Tibetan wild asses (Liu *et al.* 2022), alpine musk deer (Jiang *et al.* 2021), and wild goitered gazelles (Qin *et al.* 2020) that are found on the QTP, Firmicutes and Bacteroidetes were the two predominant taxa among PN, TA, and TS. Emerging evidence suggests that Firmicutes play a significant role in promoting plant fiber degradation and transforming cellulose into short‐chain fatty acids, thus enhancing forage digestibility and providing plenty of energy for host survival activities (Zhang *et al.* 2017; Cabral *et al.* 2022). Bacteroidetes are recognized as the optimal species for the degradation of high‐molecular‐weight organic matter, such as plant proteins and carbohydrates (Thomas *et al.* 2011). In our study, when compared to TS, the indicator phyla Firmicutes and Bacteroidetes, as well as some indicator genera such as *Prevotella* and *Bacteroides*, have a richer relative abundance in PN and TA. Studies on yaks (Guo *et al.* 2021) and Tibetan wild asses (Liu *et al.* 2022) have shown that these animals could tolerate roughage owing to abundant Firmicutes and Bacteroidetes levels. Here, the high presence of indicator species in small wild ruminants suggests that these animals might be more tolerant to roughage than TA. This speculation is supported by the positive relationship between the forage apparent nutrient digestibility and indicator microbial communities in our correlation analysis.

### Gut microbiota enterotype and functional contexts of the represented genus in the adaption to sparse vegetation

In the present study, we explored the enterotype classification and function of small wild ruminants. There is an indelible relationship between enterotype and host adaption. Studies have shown that yaks shift their enterotypes to use nitrogen and energy effectively in the cold season from November to April (Guo *et al.* 2021). Plateau pikas adapt to complex food conditions by forming different enterotypes (Fan *et al.* 2020). In our study, the same gut microbiota enterotype classification of wild animals indicated a convergent adaptation of the gut microbiota when reared in different habitats. This study thus provides biological insights into the functions and functional genomic information of these two enterotypes. Enterotype 1 mainly comprised *Bacteroides*, *Alistipes*, and *Eunacterium*. Studies have demonstrated that *Bacteroides* not only participate in the decomposing of plant cellulose, hemicellulose, and other polysaccharides but also contribute to nitrogen cycling, that is, assisting the host in metabolizing and transforming nitrogen compounds (Thomas *et al.* 2011; Batista‐García *et al.* 2016). *Alistipes* and *Eunacterium* are essential for plant polysaccharide decomposition, maintenance of the intestinal mucosal barrier function, and regulation of the immune system (Wang *et al.* 2017; Mukherjee *et al.* 2020). Therefore, we hypothesized that the Enterotype 1 cluster has a higher forage utilization efficiency than Enterotype 2; this was further confirmed by the results of the high apparent digestibility of forage (Table 2). Our study also clarified the influence of the *Bacteroides*, *Alistipes*, and *Eunacterium* enterotypes on FA and energy metabolisms. Enzymes involved in fatty acid biosynthesis and oxidative phosphorylation pathways were enriched in Enterotype 1, which indicates that this enterotype also plays a vital role in energy production and in maintaining the energy balance of the host.

### The lower abundance of pathogenic bacteria reduces the incidence of intestinal diseases in small wild ruminants

Many pathogens of the gut microbiome can cause enteropathies. Although these pathogenic bacteria require specific conditions (e.g. animal immune system deficiencies or living environment changes) to initiate infection (Ludwig *et al.* 2010; Ghaisas *et al.* 2016), analysis of the distribution characteristics of pathogenic bacteria in the intestinal flora can be an important reference base to determine the future health of the host (Gao *et al.* 2023). In this study, we used metagenomic sequencing technology to quantitatively analyze 14 potential pathogenic bacteria (Jiang *et al.* 2023). We found that 10 pathogenic bacteria, such as *Clostridium, Treponema, Streptococcus*, and *Bacillus* were significantly enriched in TS. Therefore, we speculate that as the number of potential pathogenic bacteria increases, there is a higher risk of gastrointestinal disease in TS; that is, the lower abundance of pathogenic bacteria in the gastrointestinal tract of small wild ruminants may aid them to better adapt to the challenges of intestinal diseases caused by the plateau wilderness environment.

### The abundant cellulose, protein‐decomposing bacteria, and functional genes enhanced the utilization potential of forage in the small wild ruminants

Most herbivores do not digest grasses on their own. Rather, they depend on their gut microbes to decompose plant biomass into various nutrients (Niu *et al*. 2015). The functional analysis of gut microbiota is of great significance for understanding the association between microbes and hosts, as well as nutrient digestion and absorption (Krajmalnik‐Brown *et al.* 2012). In the present study, we quantitatively analyzed several cellulolytic and proteolytic bacteria, including *R. flavefaciens*, *P. ruminicola*, *Butyrivibrio* *fibrisolvens*, *R*. *albus*, *R. amylophilus*, and *F. succinogenes* (Hastie *et al.* 2008; Liu *et al.* 2020a). Notably, these functional bacteria were significantly more abundant in TA and PN than in TS. This finding is consistent with that of a previous study on the function of the intestinal flora in Tibetan wild asses, yaks, and sheep (Liu *et al.* 2022). That is, compared to domestic animals, wild animals have rich forage‐digesting flora that can ensure the host maintains an efficient decomposition and absorption capacity for cellulose, proteins, and other nutrients when consuming low‐quality and quantity forage to adapt to harsh living environments. In the gut, the breakdown of nutrients is often accompanied by the production of hydrogen, which archaea use to produce greenhouse gases, such as methane and carbon dioxide (Hoffmann *et al.* 2013; Su *et al.* 2022). In the present study, five predominant methane‐producing archaea were enriched in the TS gut. Studies have revealed that yaks and TS were more environmentally friendly high‐altitude mammals than cattle and goats, as their unique gut microbial structure aided biological control of greenhouse gas emissions (Zhang *et al.* 2016). Although the greenhouse gases of TA and PN cannot be measured in a laboratory, we speculate that PN and TA may be low methane emission species based on the low archaea consortiums, and this energy‐efficient utilization strategy enables PN and TA to cope with the perennial cold climate of TRSNP.

Herbivores can utilize carbohydrate‐rich plant residues as substantial energy resources. Fiber‐decomposing enzymes produced by the gut microbiota are of great significance in research related to forage utilization (Gharechahi *et al.* 2021). In the present study, from the perspective of CAZymes, we found that glucoside hydrolases were the most diverse (*n* = 32763) and abundant (average 23.85% of the total), which is consistent with the findings of Cantarel *et al.* (2009). In addition, a total of 21 high relative abundance of CAZymes were associated with cellulase, hemicellulase, and esterase deconstruction (Flint *et al.* 2012; Gharechahi *et al.* 2021; Peng *et al.* 2021). Except for GH1 and CE4, 19 genes encoding cellulases, hemicellulases, and esterases were significantly more abundant in PN and TA. These discrepancies may be explained by differences in feeding niches. Wild animals living in poor conditions on TRSNP usually feed on forage with high levels of indigestible plant fiber, whereas TS selectively consumes forage of high quality under the care of herdsmen. The abundant functional CAZyme genes indicated convergent evolution of gut microbiota for host adaption and further confirmed that the wild animal gut microbiota are a worthy resource for in‐depth analysis, such as microbial enzyme preparation (Levin *et al.* 2021).

However, the mechanism of microbial‐mediated severe feeding environment adaptation of herbivores is a complex process. There are some limitations to this study, such as the difficulty of sample collection, especially that of the wild animals, resulting in a slightly decreased sample size and limited data acquisition. Additionally, the feeding habits of the experimental animals are not clear; the wild animals may balance the contradiction between nutrient demand and forage supply by eating some plants with higher nutrition. Therefore, subsequent studies on feeding habits and feeding behaviors should be conducted to explore the potential mechanisms affecting animal intestinal flora. Moreover, using metabolomics technology can provide important metabolite information when studying severe food environment adaption among different hosts; due to the limited samples, we should use metabolomics technology in future studies to shed some light on the environmental adaptation of hosts.

## CONCLUSION

The intervention of gut microbiota is one of the key factors for small wild ruminants to adapt to the food scarcity situation in the Three‐River‐Source National Park. Our results revealed that gut microbiota‐regulated herbivores, especially wild animals, can adapt to the pasture nutritional stress from their habitat by changing their microbial diversity, gut enterotypes, and functional bacterial species and genes that relate to decreasing intestinal disease occurrence and enhancing forage fiber and crude protein decomposing. This study has important implications in the research of the dietary adaptions of wild ruminants in the Three‐River‐Source National Park, China.

## CONFLICT OF INTEREST STATEMENT

The authors declare no conflict of interest.

## Supporting information


**Figure S1** Lollipop charts showing indicator species of gut microbiota at phylum (a) and genus (b) level. Lollipop are colored by different microbita.
**Figure S2** Metabolic pathway construction and enzymes that involved in oxidative phosphorylation.
**Figure S3** The rest percentages of ARGs among the three small ruminants.
**Table S1** The information of 16S rRNA amplicon sequencing.
**Table S2** The relative abundance of gut microbiota in each host at the phylum level.
**Table S3** The relative abundance of top 20 gut microbiota in each host at the genus level.
**Table S4** Topological properties of the MENs under WR or TS.
