# Supplementary material for: Multi‐omics reveal the gut microbiota‐mediated severe foraging environment adaption of small wild ruminants in the Three‐River‐Source National Park, China
Source: Integr Zool. 2024 May 2;20(5):916–35. doi: 10.1111/1749-4877.12830 (PMC12463759; doi:10.1111/1749-4877.12830)
Supplement: Supplementary file 1 — Figure S1 Lollipop charts showing indicator species of gut microbiota at phylum (a) and genus (b) level. Lollipop are colored by different microbita. Figure S2 Metabolic pathway construction and enzymes that involved in oxidative phosphorylation. Figure S3 The rest percentages of ARGs among the three small ruminants. Table S1 The information of 16S rRNA amplicon sequencing. Table S2 The relative abundance of gut microbiota in each host at the phylum level. Table S3 The relative abundance of top 20 gut microbiota in each host at the genus level. Table S4 Topological properties of the MENs under WR or TS. [file INZ2-20-916-s001.docx]

**SUPPLEMENTARY MATERIALS**

**Figure S1** Lollipop charts showing indicator species of gut microbiota at phylum (a) and genus (b) level. Lollipop are colored by different microbita. A high indicator value indicates a species has a high indicator power for corresponding group.

**
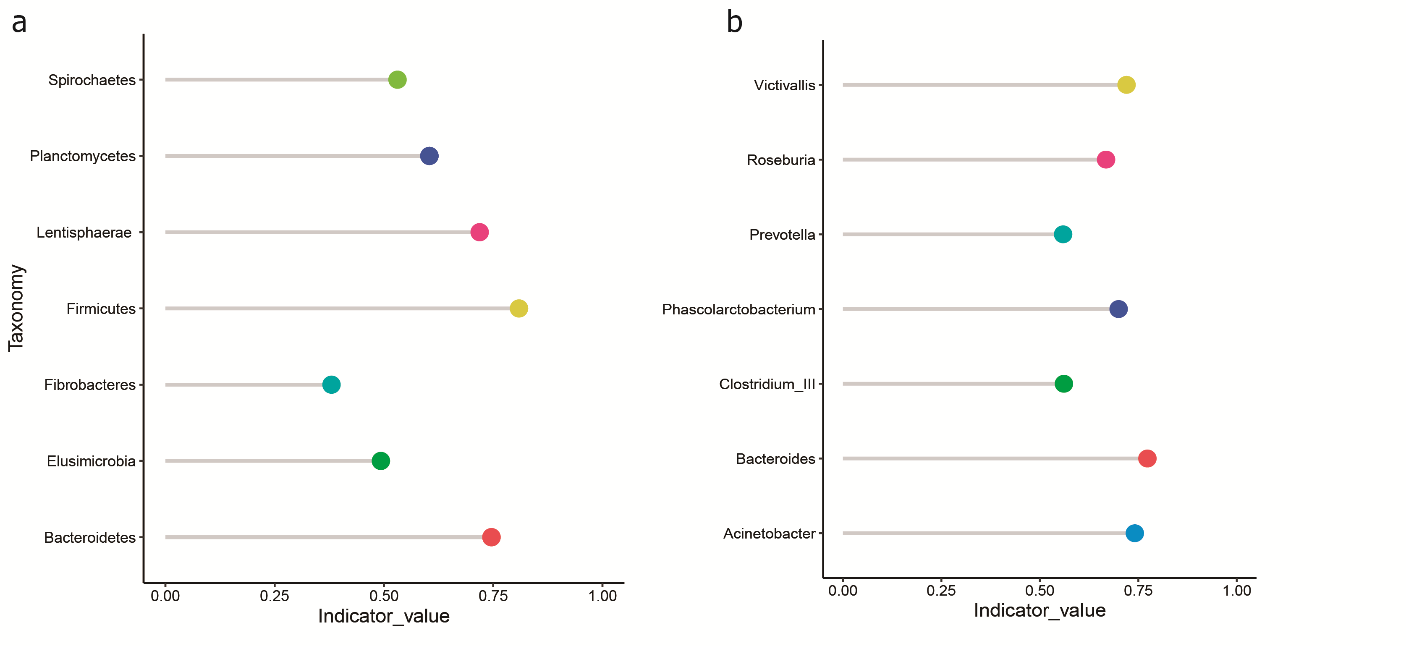
**

**Figure S2** Metabolic pathway construction and enzymes that involved in oxidative phosphorylation. (a) The metabolic pathway of oxidative phosphorylation. (b) Enzymes abundance significant analysis between E1 and E2.

**
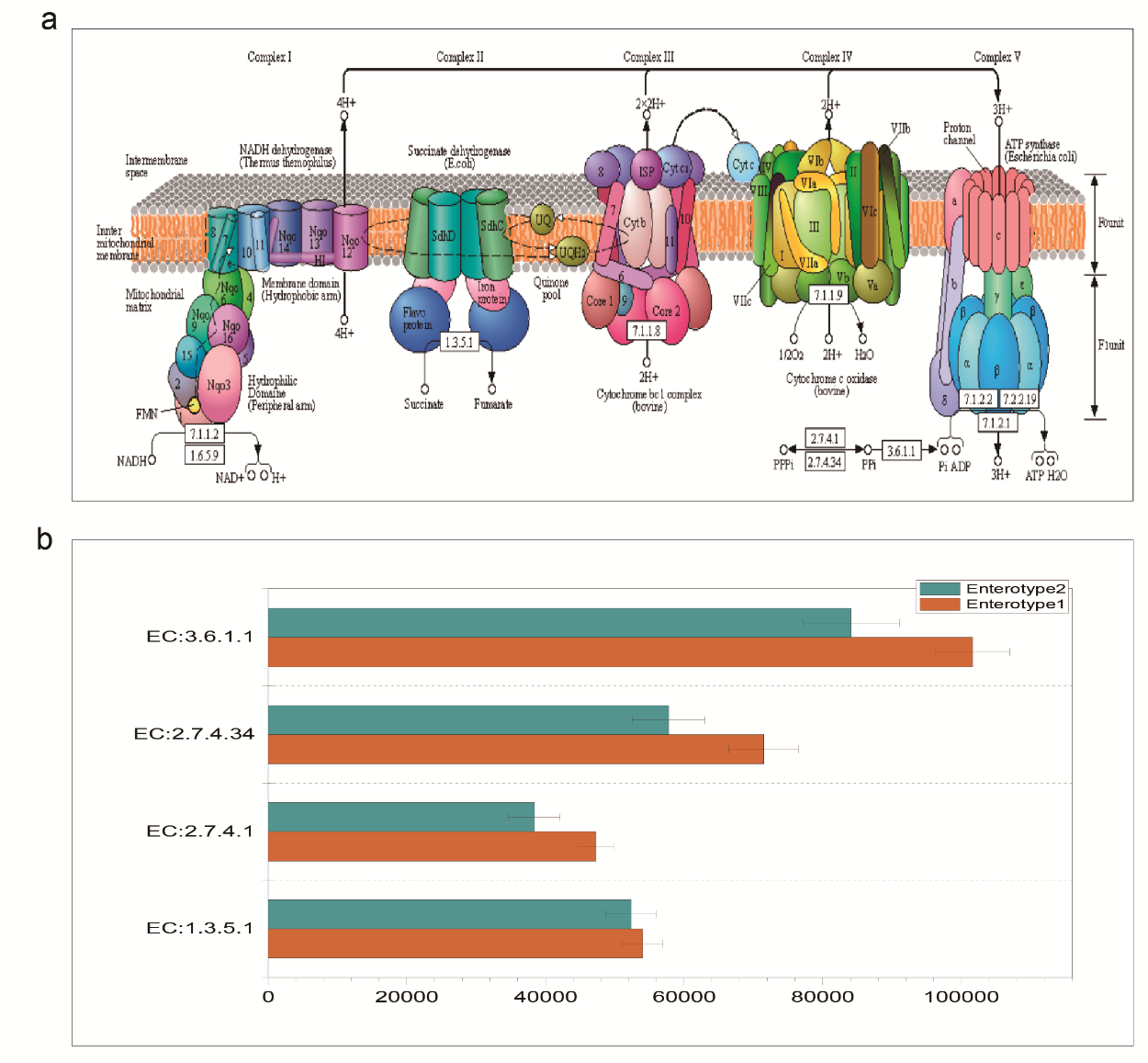
**

**Figure S3** The rest percentages of ARGs among the three small ruminants.

**
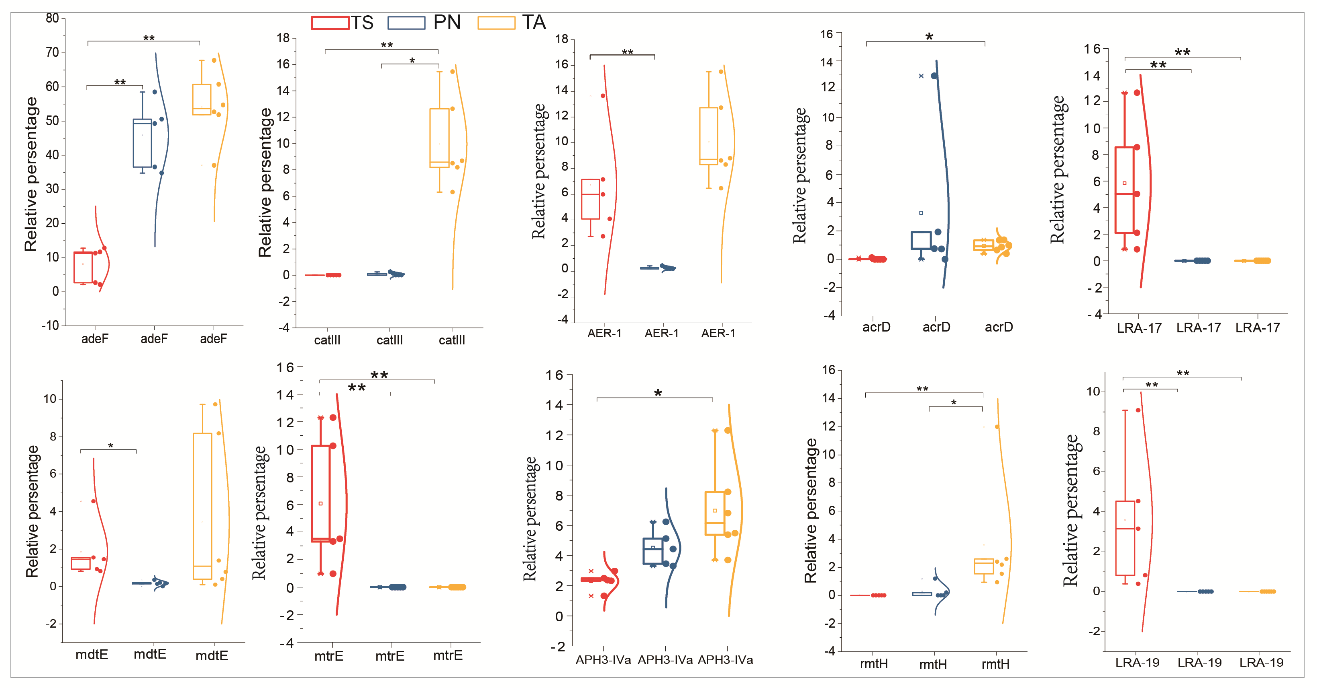
**

**Table S1** The information of 16S rRNA amplicon sequencing

| **1910442**  Reads (1.9M) |
| --- |
| 16 Samples |
| **10527 ASVs** |
| 284229 Counts |
| 205939 Count =0 (72.5%) |
| 21210 Count =1 (7.5%) |
| 28299 Count >=10 (10.0%) |
| 71 ASVs found in all samples (0.7%) |
| 192 ASVs found in 90% of samples (1.8%) |
| 1446 ASVs found in 50% of samples (13.7%) |
| Sample sizes: **min 36172**, lo 59242, med 69089, mean 70757.1, hi 80206, max 126686 |

**Table S2** The relative abundance of gut microbiota in each host at the phylum level

| Phylum | PN | S | TA | **Average(%)** |
| --- | --- | --- | --- | --- |
| Firmicutes | 63.34±1.30 | 39.04±3.20 | 46.67±2.18 | 47.84±2.73 |
| Bacteroidetes | 17.16±0.80 | 6.97±1.14 | 12.50±1.27 | 11.98±1.11 |
| Proteobacteria | 1.11±0.29 | 1.98±0.4 | 3.74±2.19 | 2.31±0.76 |
| (Unassigned) | 2.8±0.36 | 1.40±0.2 | 2.69±0.53 | 2.17±0.26 |
| Actinobacteria | 0.29±0.03 | 1.05±0.14 | 1.80±1.38 | 1.09±0.46 |
| Planctomycetes | 3.81±0.77 | 25.86±5.59 | 15.68±2.16 | 16.84±3.2 |
| Verrucomicrobia | 6.38±1.87 | 13.80±3.13 | 19.96±4.85 | 13.79±2.39 |
| Candidatus_Saccharibacteria | 0.56±0.13 | 0.75±0.14 | 0.43±0.12 | 0.60±0.08 |
| Acidobacteria | 0.02±0.01 | 0.89±0.35 | 0.91±0.79 | 0.66±0.30 |
| Lentisphaerae | 3.03±1.03 | 0.03±0.01 | 0.15±0.04 | 0.85±0.40 |
| candidate_division_WPS-1 | 0.00±0.00 | 0.87±0.38 | 0.19±0.11 | 0.43±0.18 |
| Euryarchaeota | 0.62±0.47 | 0.84±0.6 | 0.11±0.03 | 0.55±0.29 |
| Spirochaetes | 0.28±0.16 | 0.58±0.13 | 0.05±0.02 | 0.34±0.09 |
| Tenericutes | 0.12±0.03 | 0.08±0.02 | 0.03±0.01 | 0.07±0.01 |
| Gemmatimonadetes | 0.00±0.00 | 0.21±0.13 | 0.37±0.33 | 0.21±0.12 |
| Elusimicrobia | 0.16±0.08 | 0.03±0.01 | 0.03±0.01 | 0.06±0.03 |
| Chloroflexi | 0.00±0.00 | 0.07±0.05 | 0.11±0.10 | 0.07±0.04 |
| candidate_division_WPS-2 | 0.00±0.00 | 0.01±0.01 | 0.07±0.05 | 0.02±0.02 |
| Fibrobacteres | 0.28±0.25 | 0.00±0.00 | 0.00±0.00 | 0.07±0.07 |
| Parcubacteria | 0.01±0.00 | 0.00±0.00 | 0.01±0.01 | 0.01±0.00 |
| Deferribacteres | 0.01±0.01 | 0.01±0.00 | 0.00±0.00 | 0.01±0.00 |

**Table S3** The relative abundance of top 20 gut microbiota in each host at the genus level

| Genus | PN | S | TA | **Average** |
| --- | --- | --- | --- | --- |
| Akkermansia | 5.70±1.92 | 12.84±2.87 | 6.20±4.91 | 8.23±1.75 |
| Bacteroides | 6.59±0.45 | 3.43±0.6 | 2.90±0.46 | 4.09±0.47 |
| Phascolarctobacterium | 0.89±0.04 | 0.86±0.29 | 2.4±0.40 | 1.35±0.24 |
| Alistipes | 1.38±0.11 | 1.49±0.30 | 0.83±0.15 | 1.25±0.15 |
| Clostridium_XlVa | 1.62±0.21 | 0.97±0.15 | 1.23±0.08 | 1.22±0.11 |
| Eubacterium | 1.45±0.31 | 0.99±0.16 | 1.17±0.27 | 1.17±0.14 |
| Ruminococcus | 1.01±0.06 | 0.86±0.13 | 1.26±0.21 | 1.02±0.10 |
| Oscillibacter | 1.14±0.06 | 0.92±0.10 | 1.02±0.19 | 1.01±0.08 |
| Victivallis | 2.99±1.02 | 0.03±0.01 | 0.12±0.04 | 0.84±0.40 |
| Clostridium_IV | 0.57±0.04 | 0.38±0.06 | 1.42±0.89 | 0.76±0.30 |
| Saccharibacteria_genera_incertae_sedis | 0.56±0.13 | 0.75±0.14 | 0.43±0.12 | 0.60±0.08 |
| Weissella | 0.01±0.00 | 0.01±0.00 | 1.88±1.70 | 0.60±0.57 |
| Flavonifractor | 0.39±0.02 | 0.7±0.11 | 0.54±0.08 | 0.57±0.06 |
| Clostridium_III | 0.91±0.20 | 0.27±0.05 | 0.59±0.11 | 0.54±0.09 |
| WPS-1_genera_incertae_sedis | 0.00±0.00 | 0.87±0.38 | 0.19±0.11 | 0.43±0.18 |
| Methanobrevibacter | 0.22±0.16 | 0.81±0.60 | 0.06±0.02 | 0.42±0.27 |
| Marinobacter | 0.02±0.00 | 0.59±0.36 | 0.46±0.14 | 0.40±0.17 |
| Prevotella | 0.02±0.00 | 0.07±0.30 | 0.74±0.04 | 0.34±0.15 |
| Sphingomonas | 0.01±0.00 | 0.06±0.03 | 0.99±0.88 | 0.34±0.29 |
| Treponema | 0.28±0.16 | 0.58±0.13 | 0.05±0.02 | 0.34±0.09 |
| Unclassfied | 68.94±0.8 | 66.69±3.77 | 68.65±5.02 | 68.09±1.49 |

**Table S4** Topological properties of the MENs under WR or TS

|  | | WR | TS |
| --- | --- | --- | --- |
| Numbers of ASVs* | | 2601 | 1156 |
| Empirical network | Similarity threshold | 0.95 | 0.95 |
|  | Total nodes | 677 | 407 |
|  | Total links | 1094 | 420 |
|  | R square of power law | 0.92 | 0.92 |
|  | Average Connectivity (avgK) | 3.232 | 2.064 |
|  | Average clustering coefficient (avgCC) | 0.032 | 0.055 |
|  | Average path distance (GD) | 6.538 | 3.83 |
|  | Harmonic geodesic distance (HD) | 5.148 | 2.578 |
|  | Geodesic efficiency (E) | 0.194 | 0.388 |
|  | Connectance (Con) | 0.325 | 0.023 |
|  | No. of modules in the largest connected component | 30 | 16 |
|  | No. of modules | 116 | 92 |
|  | No. of large modules** | 21 | 18 |
|  | No. of nodes in large modules** | 386 | 210 |
|  | Percentage of nodes in large modules (%) ** | 57 | 52 |
|  | Positive links | 714 | 228 |
|  | Negative links | 380 | 192 |
|  | Proportion (Positive/total) | 65.26 | 54.28 |

*The total numbers of ASVs with non-zero sequence numbers in at least half of samples used in each network.

**Large modules contain at least 5 nodes.
